# Porcine Circovirus-Like Virus P1 Inhibits Wnt Signaling Pathway *in Vivo* and *in Vitro*

**DOI:** 10.3389/fmicb.2018.00390

**Published:** 2018-03-12

**Authors:** Xuejiao Zhu, Libin Wen, Shaoyang Sheng, Wei Wang, Qi Xiao, Meng Qu, Yiyi Hu, Chuanmin Liu, Kongwang He

**Affiliations:** ^1^Institute of Veterinary Medicine, Jiangsu Academy of Agricultural Sciences – Key Laboratory of Veterinary Biological Engineering and Technology, Ministry of Agriculture, Nanjing, China; ^2^Jiangsu Co-innovation Center for Prevention and Control of Important Animal Infectious Diseases and Zoonoses, Yangzhou, China; ^3^Jiangsu Key Laboratory for Food Quality and Safety – State Key Laboratory Cultivation Base of Ministry of Science and Technology, Nanjing, China

**Keywords:** porcine circovirus-like virus P1, PMWS, inhibit effect, VP1, Wnt signaling

## Abstract

Porcine circovirus-like virus P1 is an important pathogen of the current pig industry, the infection mechanism is not entirely clear. Wnt signaling pathway plays an important role in the growth of young animals and infection of some viruses. This study was designed to demonstrate the effects of P1 infection on the Wnt signaling pathway. *In vivo* experiments, we demonstrated the down-regulatory effects of P1 infection in piglets and mice on the downstream components expression levels of Wnt signaling pathway, and the effects of Wnt signaling pathway activation on the pathogenesis of P1. *In vitro* studies, we found P1 infection down-regulated protein level of β-catenin and mRNA level of mmp2, prevented the β-catenin from entering into nucleus, abolished the TCF/LEF promoter activity, proved that P1 could inhibit the activation of Wnt signaling pathway *in vitro*. Finally, we found that VP1 of P1 virus also had the inhibitory effects on Wnt signaling pathway *in vitro*, elucidated the mechanism of P1’s inhibitory effects on the Wnt signaling pathway and offered the possibility that the suppression of Wnt signaling pathway was involved in the post-weaning multisystemic wasting syndrome (PMWS), laying a foundation for elucidating the pathogenesis of P1.

## Introduction

Porcine circovirus like virus is one of the recently discovered minimal genomic DNA viruses (648 nucleotides). The discovery of the virus occurred on the diagnosis of porcine circovirus type 2 (PCV2) in the tissue sample of post-weaning multisystemic wasting syndrome (PMWS). The porcine circovirus-like virus shares similar sequence with the PCV2 ORF2 (opening read frame 2) sequence, named porcine circovirus-like virus P1 and P2 (Wen et al., 2011). Wen successfully constructed P1, P2 single-copy molecular cloning and double-copy tandem molecular cloning, after transfection into PK-15 cells, inclusion bodies were found in the cytoplasm and nucleus. During continuous passage of five generations, P1 and P2 agents can be detected by PCR in the cells transfected with P1, P2 double-copy tandem molecular cloning (Wen et al., 2012a,b,c). At present, there are few studies on P1. P1 has a single-stranded circular DNA genome and is highly homologous to PCV2 ORF. P1 is thought to be a recombinant virus formed by PCV2 and another virus (Wen et al., 2008), which also contains three main reading frames: ORF1, ORF2, and ORF3. Recently, it was reported that porcine circovirus-like virus predominantly encoded about 12.5 kDa of structural protein, namely, VP1 protein which was expressed by ORF1. Homology analysis of the N-terminal amino acid sequence of ORF1 showed that P1 ORF1 was highly homologous to the N-terminal domain of PCV2 ORF2. However, the C-terminal region has a low homology because the P1 ORF1 reading frame has a nucleotide shift. P1 has a close relationship with PCV2, both of which have similar structural and biological characteristics (Wen et al., 2015).

Epidemiological studies have confirmed that porcine circovirus-like virus is prevalent in Chinese pig farms (Wen et al., 2011, 2017a,b). *In vivo* studies have shown that porcine circovirus-infected pigs displayed PMWS (Wen et al., 2012d). Thus, P1 infection may also lead to pig immunosuppression, concurrent or secondary to other pathogens, causing serious economic loss in the pig industry. However, the replication mechanisms and pathogenicity of P1 are not entirely clear. Thus, it is important to understand the mechanism of which P1 causes PMWS and to explore the causes of weight loss of piglets in clinical.

The Wnt pathway is a key signaling pathway in the growth of the body, capable of controlling cell polarity and differentiation, involving in many diseases, including cancer, diabetes, musculoskeletal and kidney disorders (Clevers and Nusse, 2012; Anastas and Moon, 2013). The pathway involves a variety of receptors, ligands and effect molecules with complex positive and negative feedback loops. The Wnt signaling pathway is divided into β-catenin dependent signaling and β-catenin independent signaling pathway. Although these two routes have different signal cascades, they share a cross reaction. β-catenin dependent signaling pathway is often at the “off” state. When the Wnt ligand binds to frizzled protein (FZD) receptor, the pathway is activated. The activation of this pathway requires low-density lipoprotein receptor-related proteins 5, 6 (LRP5 and LRP6) and enrichment of Disheveled proteins (DVL) (Voronkov and Krauss, 2013). A destruction complex consists of APC protein (adenomatous polyposis coli), AXIN (axonal protein) and GSK-3β (glycogen synthase kinase-3β) phosphates. β-catenin leads to its ubiquitination, the proteasome is destroyed, and cytoplasmic β-catenin transferred to the nucleus. The binding of β-catenin in the nucleus to TCF and LEF transcription factors leads to the activation of these transcription factors, thereby activating the Wnt signaling pathway. Thus, the increase of β-catenin in nucleus leads to the activation of the Wnt pathway, which activates the downstream gene expressions of Wnt, such as myc, cyclin-D1 (CCND1) and mmps, which regulate differentiation and proliferation of cells (Clevers and Nusse, 2012; Anastas and Moon, 2013). The activation of TCF/LEF-1 and accumulation of β-catenin in the nucleus were as typical markers for activation of β-catenin dependent signaling pathway. In addition, viruses and Wnt signaling pathway are also inseparable. Viruses can often alter Wnt signaling pathways for their own purpose in many ways. For examples, LMP1 protein of Epstein–Barr virus (EBV) inhibited SIAH1 gene expression in B lymphoma cells by up-regulating β-catenin expression and increase the accumulation of β-catenin in the nucleus (You et al., 2011). Hepatitis C virus (HCV) core protein utilized SFRP1 (secreted frizzled related protein) (Quan et al., 2014) to up-regulate the expression of Wnt1 and WISP1 (Wnt1-induced signaling pathway protein-1), and target protein in Wnt signaling pathways (Fukutomi et al., 2005; Liu et al., 2011a,b). HCV non-structural protein NS5A can promote tumor growth by activating Wnt signaling pathway (Liu et al., 2011a), HCV also could activate the myc promoter which stabilizes β-catenin and activates the Wnt signaling pathway (Street et al., 2005; Park et al., 2009; Higgs et al., 2013). In addition, it has been reported that lithium chloride (LiCl) is able to activate the Wnt signaling pathway and inhibit the inflammatory response, thereby inhibiting the infection of porcine reproductive and respiratory syndrome (PRRS) virus (Hao et al., 2015).

P1 can cause PMWS, the principal manifestations of PMWS in clinical is weight loss. At present, there is no report on the effect of P1 on Wnt signaling pathway. In this study, the effect of P1 on Wnt signaling pathway *in vitro* and *in vivo* were clarified. The relationship between Wnt signaling pathway and PMWS was investigated by *in vivo* experiment.

## Materials and Methods

### P1 Virus

Molecular cloning of two copies of P1 strain (NCBI serial number: EF514716) genome were firstly ligated into the pSK vector by our lab (Wen et al., 2012d), and the infectious virus was obtained by five passage of generations in the ST cells and preserved at -70°C by our lab.

### Materials

pRL-TK and pGL-Basic-6 were purchased from Invitrogen company, pGL-TCF/LEF and pLV-VP1-mcherry were prepared by our lab. Dual-luciferase system was purchased from Promega (Madison, WI, United States). All the antibodies were purchased from Beyotime (China) except that anti-Dickkopf-1 was purchased from Santa Cruz (CA, United States). Nucleus and cytoplasm separation kit was purchased from Beyotime. ST cells (American Culture Collection, Manassas, VA, United States) were grown in Dulbecco’s modified Eagle’s medium supplemented with 10% fetal calf serum, 250 U/mL penicillin, and 250 μg/mL streptomycin.

### *In Vivo* Experiment

Animals: Ten 3-week healthy piglets free of P1, PRRSV, PRV, and PCV2 were randomly divided into two groups, one group was as control group, one group was challenged by P1 infectious virus. Blood samples were obtained and detected for antibodies and pathogens prior to the animal experiment. The antibodies against PCV2 and P1 were detected by indirect-ELISA methods set up by our laboratory, the antibodies against PRV gI and PRRSV were detected by IDEXX (United States) ELISA kits. The pathogens were detected by PCR method. Piglets were challenged with P1 infectious virus by intramuscular inoculation, the challenge dose was 4 mL (TCID_50_ 10^∧^5/mL) per pig. Clinical symptoms, body temperature and food consumptions were observed every day. At 5 weeks post-infection, all piglets were euthanized and biopsied. Tissues were equally collected of each piglet for grinding and homogenate. DNA and RNA were extracted from each homogenate sample after three times freezing and thawing. Take the same amount of RNA for reverse transcription. DNA and cDNA were preserved at -70°C. Weight gains at 35 days post-infection (dpi) were determined and expressed as relative average daily weight gains (ADWG) [(body weight at 35 dpi - body weight at 0 dpi)/body weight at 0 dpi/35].

Forty ICR mice were randomly divided into four groups, 10 for each group, two groups of mice were inoculated with 200 μL P1 infectious virus, and other two groups were inoculated with sterilized PBS as control group. At 3 weeks post-infection, each mouse of challenge and control groups was intraperitoneal injected with LiCl (4 mmol/kg) every day, other two groups were injected the same dose of PBS. Autopsy was executed at 4 weeks post-infection. The tissues were equally collected of each mouse for grinding and homogenate; the DNA and RNA were extracted from each homogenate sample after three times freezing and thawing. Take the same amount of RNA for reverse transcription. DNA and cDNA were preserved at -70°C for quantitative PCR. Weight gains from 28 days (4 weeks) post-infection (dpi) compared to 21 dpi (3 weeks) were determined and expressed as relative average daily weight gains (ADWG) [(body weight at 28 dpi - body weight at 21 dpi)/body weight at 21 dpi/7]. By analogy to the formula, the weight gains were measured and calculated at each week since 2 wpi.

All animal experimental protocols were approved by the Institutional Animal Care and Ethics Committee of Jiangsu Academy of Agricultural Sciences (NKYVET 2015-0126) and met the standards of the International Guiding Principles for Biomedical Research Involving Animals.

### Quantitative PCR

P1 viral loads distributions in organs were detected. Take the same amount of RNA for reverse transcription by random hexamers with reverse transcription kit (Vazyme, China), reverse transcription RNA was 500 ng in total. The RNA reverse transcription products of liver, spleen, lung, kidney, heart, and inguinal lymph node were subjected to quantitative PCR. The relative expression of matrix metalloproteinases (mmp) 2, mmp7, and mmp9, DKK-1, c-myc, and cyclin D1 (CCND1) were detected (primers were listed in **Table 1** and the concentration was 10 μM). The SYBR Green Real-time PCR Mix (Takara, Japan) was used according to the manufacturer’s recommendations. The reaction procedure was as follows: 95°C for 15 s, followed by 40 cycles at 95°C for 5 s and 60°C for 34 s. Data analysis was conducted using the 2^-ΔΔCt^ comparative threshold method, and gene expression was normalized to the level of β-actin mRNA. The qPCR was performed on an ABI PRISM 7500 (Applied Biosystems, Foster City, CA, United States). The results are presented as fold-change of relative expression compared with the mock group.

**Table 1:** Primers for qPCR.

Primer	Sequences
P1-RT-F	AAGACCCCCCACTTAAACCC
P1-RT-R	GAGAGGCGGGTGTTGAAGAT
β-actin-RT-F (swine)	CTCTTCCAGCCCTCCTTCCT
β-actin-RT-R (swine)	ACGTCGCACTTCATGATCGA
mmp2-RT-F (swine)	ACACCTATACCAAGAACTTCCG
mmp2-RT-R (swine)	TGTCCGCCAGATGAACCG
mmp7-RT-F (swine)	TTTCGCCTGCCTATAACTGGA
mmp7-RT-R (swine)	TTTGGCTGGCTTGGGAATAG
mmp9-RT-F (swine)	CCACAGGCCCTCCTTCAG
mmp9-RT-R (swine)	TGAACAGCAGCACCTTACC
c-myc-RT-F (swine)	CTCGGACTCTCTGCTCTCCT
c-myc-RT-R (swine)	TTGTTCTTCCTCAGAGTCGCT
DKK-1-RT-F (swine)	AGGGGAAATTGAGGAAACCA
DKK-1-RT-R (swine)	GGCACAGTCTGATGATCGGA
CCND-1-RT-F (swine)	GCCCTCCGTGTCCTACTTCA
CCND-1-RT-R (swine)	AGACCTCCTCCTCGCACTTCT
β-actin-RT-F (mouse)	CATCCGTAAAGACCTCTATGCCAAC
β-actin-RT-R (mouse)	ATGGAGCCACCGATCCACA
mmp2-RT-F (mouse)	AGAACTTCCGATTATCCCATGATGA
mmp2-RT-R (mouse)	TGACAGGTCCCAGTGTTGGTG
mmp7-RT-F (mouse)	GGCGGAGATGCTCACTTTGAC
mmp7-RT-R (mouse)	AATTCATGGGTGGCAGCAAAC
mmp9-RT-F (mouse)	CTTCCAACTTTGACAGCGACA
mmp9-RT-R (mouse)	GGAGTGATCCAAGCCCAGTG
c-myc-RT-F (mouse)	TCAAGAGGTGCCACGTCTCC
c-myc-RT-R (mouse)	TCTTGGCAGCAGGATAGTCCTT
DKK-1-RT-F (mouse)	GCGGCAAGACCTACACCAAGAG
DKK-1-RT-R (mouse)	CTTTCGGTAGTGGCGGGTAAGC

### Nucleus and Cytoplasm Separation Experiment

ST cells were infected by P1 infectious virus or mock infected (ST cells lysate) for 48 h, or ST cells were transfected with pLV-VP1-mcherry or mock for 24 h. LiCl at a final concentration of 70 mM were added into culture (as motivation control) or not 24 h prior to harvest for nucleus and cytoplasm separation. Cells were collected at 72 hours post-infection (hpi) for nucleus and cytoplasm separation following the manufacturer’s protocol (Beyotime, China) and then subjected to Western blot analysis. The bands were normalized with respect to β-actin (cytoplasm) and Histone H3 (nucleus).

### Luciferase Reporter Assay

ST cells transfected with pGL-TCF/LEF or pGL6 (negative control) were inoculated with P1 infectious virus or ST cell lysate to test whether pGL-TCF/LEF activation could be abolished by P1. Cells in 24-well plates were transfected using lipofectamine 3000 reagent (Invitrogen) with either 1 μg pGL-TCF/LEF or pGL6 together with 0.1 μg pRL-TK. At 24 h post-transfection, the cells were mock infected (ST cell lysate) or infected with P1 infectious virus. At 24 hpi, LiCl at a final concentration of 70 mM were added into culture (as motivation control) or not 24 h prior to harvest for luciferase assay analysis. Cell extractions were collected at 48 hpi. Luciferase activity was monitored using a dual-luciferase reporter assay system following the manufacturer’s protocol (Promega). The values were normalized with respect to Renilla luciferase activity. The results were indicated as fold-change of relative luciferase activities compared with the mock-treated group.

### Western Blot

ST cells were lysed by lysis buffer and the protein concentration was determined with the BCA Protein Assay Kit (Pierce, Thermo Fisher Scientific). Equivalent amounts of cell lysate protein (25 μg) were subjected to 12% sodium dodecyl sulfate–polyacrylamide gel electrophoresis and transferred to 0.22-μm nitrocellulose membranes (Pall, Port Washington, NY, United States). Then, the membranes were incubated with correspondent antibodies at 4°C overnight. After washing three times with phosphate-buffered saline (PBS) containing 0.05% Tween 20, the membranes were incubated at room temperature for 2 h with horseradish peroxidase-conjugated goat anti-mouse IgG or goat anti-rabbit IgG. Detection was performed using chemiluminescence luminal reagents (Thermo Fisher Scientific).

### Statistical Analysis

Statistical analysis was performed using GraphPad Prism version 5.0.2 (Software, San Diego, CA, United States). Comparisons between groups were performed using paired *t*-tests and one-way analysis of variance. *P* < 0.05 represents a statistically significant difference. All data are expressed as the mean ± standard deviation (SD).

## Results

### P1 Inhibits Wnt Signaling Pathway *in Vivo*

After challenged with P1 infectious virus, five piglets displayed obvious clinical symptoms, such as weight loss, mild pyrexia, rough hair coat, lethargy. The relative average weight gain in challenge group was 0.03859 ± 0.00479 kg, while ADWG was 0.06004 ± 0.00970 kg in control group, indicating that weight losses were significantly decreased in challenge group compared to control group.

**FIGURE 1:**
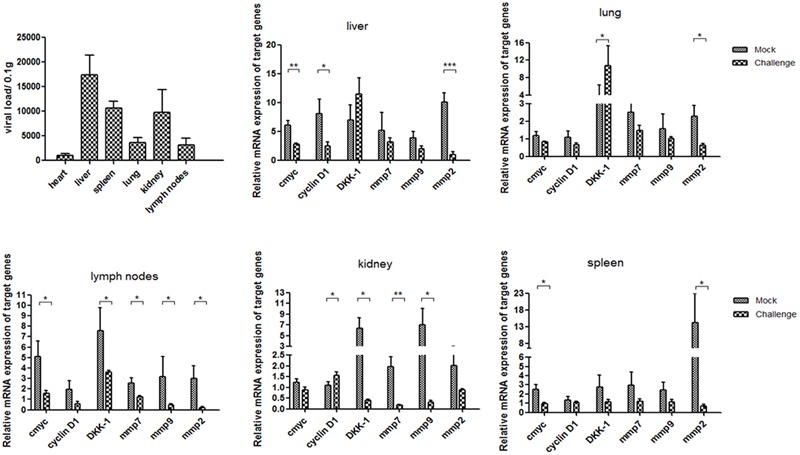
P1 inhibits Wnt signaling pathway in piglets. Distributions of viral loads of P1-inoculated piglets at 5 weeks post-challenge were measured by quantitative PCR. Endogenous c-myc, cyclin D1, DKK-1, mmp7, mmp9 and mmp2 mRNA levels were measured in the tissues from P1-inoculated piglets and mock group at 5 weeks post-challenge by quantitative PCR (*n* = 5). Statistical data were analyzed by paired *t*-tests (^∗^*P* < 0.05, ^∗∗^*P* < 0.01, ^∗∗∗^*P* < 0.001). All data are expressed as the mean ± SD.

Average day weight gain indicates average daily weight gains of piglets at 5 weeks post-infection in pig experiment. All piglets were euthanized and necropsied. Pathological examination showed that all piglets in the challenged group had mild to moderate gross lesions, histological observation showed the lungs had mild to moderate thickening of the alveolar walls, decreased alveolar space, and increased amounts of inflammatory exudate (Supplementary Figure S1). The piglets were diagnosed with PMWS. QPCR analysis showed that P1 DNA was present in the tissues challenge group at 5 weeks post-infection. The viral loads were mostly distributed in livers and reached 1.7^∗^10^∧^5 per 0.1 g, and then spleens and kidneys. Less viral loads were detected in lungs, but still could reach 4000 per 0.1 g. Minor viral loads were detected in lymph nodes and hearts (**Figure 1**).

Endogenous mmp2 and c-myc mRNA levels significantly decreased in the tissues at 5 weeks post-challenge in the challenge group (*P* < 0.05) (**Figure 1**), which significantly differed from those in the mock infected group (**Figure 1**). The levels of cyclin D1, mmp7 and mmp9 mRNA in livers and spleens of the P1 infection group did not change significantly, but significantly decreased in kidneys and livers compared with those in the mock infected group. Interestingly, the levels of DKK-1 mRNA in the P1 infection group of lung and spleen significantly increased compared to those in the mock infected group, while decreased significantly in lymph nodes and livers. DKK-1 inhibited the Wnt signaling pathway and was also the downstream component of Wnt signaling pathway; thus, its different expression and distribution suggest the different roles in Wnt signaling pathway. In all, the expression of downstream components of Wnt signaling pathway decreased indicated that Wnt signaling pathway was inhibited *in vivo*.

**FIGURE 2:**
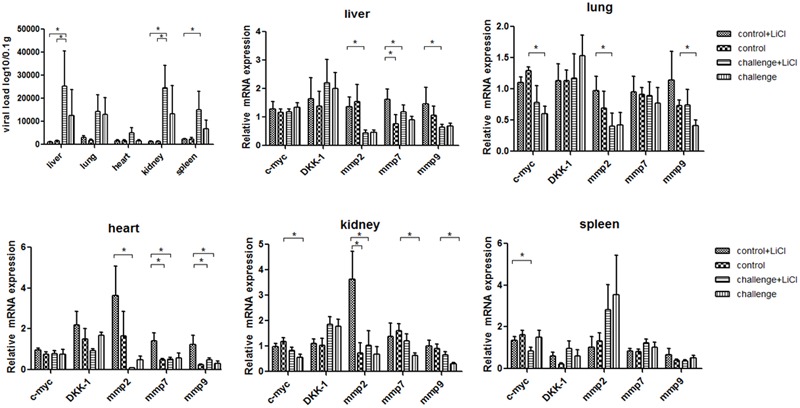
P1 inhibits Wnt signaling pathway in mice. Viral loads of tissues from P1-inoculated mice and mock group at 4 weeks post-challenge were measured by quantitative PCR. Endogenous c-myc, DKK-1, mmp2, mmp7, mmp9 mRNA levels were measured in the tissues from P1-inoculated mice and mock group at 4 weeks post-challenge by quantitative PCR (*n* = 5). Statistical data were analyzed by paired *t*-tests (^∗^*P* < 0.05). All data are expressed as the mean ± SD.

For mice experiment, the mice inoculated with P1 infectious virus displayed weight losses at 3 weeks post-infection compared with those at 2 weeks post-infection (**Table 2**), while ADWG at each week was not significantly different between challenge groups and control groups (Supplementary Table S1). The viral loads were mainly distributed in livers, kidneys, and spleens, less viral loads were detected in lungs and hearts (**Figure 2**). Surprisingly we found the challenge group stimulated by LiCl had higher viral loads than the other challenge group all though not significant (**Figure 2**).

**Table 2:** Average day weight gain in mice experiment.

ADWG (g)					
**3 wpi compare with 2 wpi**		**4 wpi compare with 3 wpi**		**4 wpi compare with 2 wpi**	
**Control**	**Challenge**	**Control**	**Challenge**	**Control**	**Challenge**

0.00289 ± 0.00241^a^	0.00143 ± 0.00283^b^	0.00472 ± 0.00425	0.00446 ± 0.00354	0.00384 ± 0.00222	0.00296 ± 0.00189

*ADWG indicates average daily weight gains in mice experiment. Different letters (a and b) indicate mutually statistically significant differences (*P* < 0.05). Data are presented as the mean ± SD.*

The function of LiCl was limited in mice, Wnt signaling pathway components mmps increased significantly only in the tissue like liver, heart, and kidney in mock group stimulated by LiCl compared with mock group. Endogenous mmp2 mRNA levels significantly decreased in the tissues at 4 weeks post-challenge in the challenge group (*P* < 0.05) (**Figure 2**), which differed significantly from those in the mock infected group except that in spleens (**Figure 2**). The levels of mmp7 and mmp9 mRNAs in the P1 infection group did not change significantly compared to those in the mock infected group in spleens, while significantly decreased in kidneys, hearts, and livers. Endogenous c-myc mRNA levels significantly decreased in the challenge group especially in the tissues like lungs, spleens, and kidneys. Interestingly, the LiCl stimulated group had higher mRNA expression of mmps and c-myc as LiCl activated Wnt signaling pathway *in vivo*, while the effects of P1 on inhibiting Wnt signaling pathway was amplified by LiCl according to the results that mRNA expression of mmps were significantly lower in the challenge group stimulated by LiCl compared with those in control group stimulated by LiCl in the tissues like livers and hearts. In all, the expression of downstream components of Wnt signaling pathway decreased indicated that Wnt signaling pathway was inhibited *in vivo*.

### P1 Inhibits Wnt Signaling Pathway *in Vitro*

#### P1 Inhibits Crucial Downstream Components Expressions of Wnt Signaling Pathway

**FIGURE 3:**
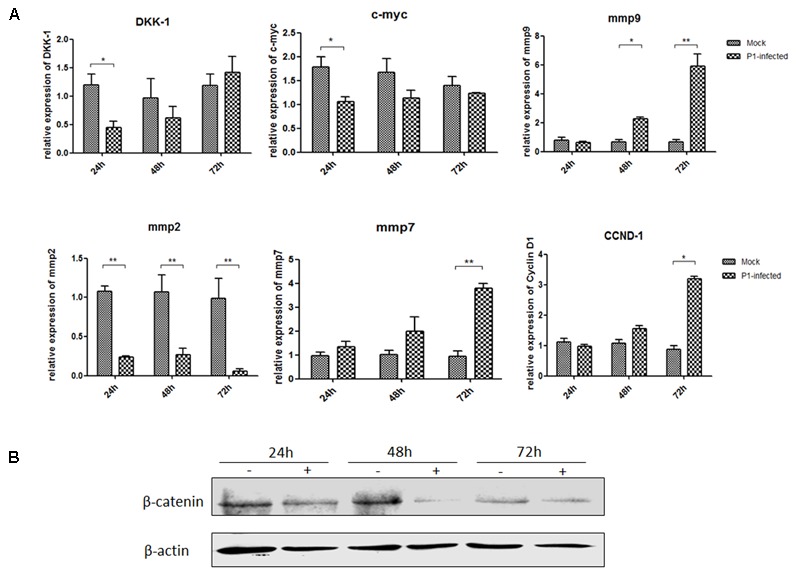
P1 inhibits crucial downstream components expressions of Wnt signaling pathway. ST cells were infected with P1 infectious virus or ST cell lysate for 24, 48, and 72 h. At each time point, ST cells were lysed and RNA was extracted. **(A)** mRNA levels of endogenous c-myc, DKK-1, mmp2, mmp7, mmp9, CCND-1 **(B)** and protein expressions of β-catenin were analyzed. The mRNA or protein expression levels of P1-infected cells are presented relative to those of mock-infected cells. The band intensity was normalized to that of β-actin. Each experiment was performed three times, and the results are representative of one independent experiment. Statistical data were analyzed by one-way analysis of variance (^∗^*P* < 0.05, ^∗∗^*P* < 0.01). All data are expressed as the mean ± SD.

ST cells were infected with P1 infectious virus for 24, 48, and 72 h. Endogenous mmp2, mmp7, mmp9, DKK-1, c-myc and CCND-1 mRNA and β-catenin protein expression levels were detected in P1-infected ST cells at 24, 48, and 72 h post-infection and compared with those in uninfected cells. Mmp2 mRNA expression level was significantly decreased in P1-infected cells at each time point (*P* < 0.05) and c-myc mRNA expression was significantly decreased only at 24 h post-infection in P1-infected cells. Surprisingly, mmp7, mmp9 and CCND-1 mRNA levels were increasing and significantly higher than those in uninfected cells at some time points, indicating that other signaling pathways might interfere the Wnt signaling pathway *in vitro* (**Figure 3A**). It was pity that there was no effective antibody for detecting P1 infectious virus, while the protein expression of β-catenin significantly decreased in P1-infected cells compared to those in uninfected cells (**Figure 3B**). As β-catenin is the critical component in Wnt signaling pathway, its accumulation finally led to the activation of the Wnt signaling pathway. Thus, it indicated that Wnt signaling pathway was inhibited by P1 infection *in vitro* though might interfered by other signaling pathways.

#### P1 Prevents β-Catenin Entering Into the Nucleus

**FIGURE 4:**
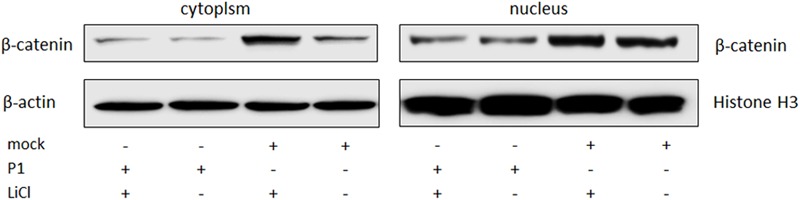
P1 prevents β-catenin entering into the nucleus. ST cells were infected by P1 infectious virus or ST cells lysate for 48 h, LiCl at a final concentration of 70 mM were added into culture (as motivation control) or not 24 h prior to harvest for nucleus and cytoplasm separation. Cells were collected at 72 hpi for nucleus and cytoplasm separation and then subjected to Western blot analysis. The bands were normalized with respect to β-actin (cytoplasm) and Histone H3 (nucleus).

ST cells were infected by P1 infectious virus or mock infected (ST cells lysate) for 48 h, and then were treated with LiCl or not at a final concentration of 70 mM 24 h prior to harvest for nucleus and cytoplasm separation. Cell nucleus and cytoplasm separation was done by the separation kit and followed the manufacturer’s instruction (Beyotime, China). Thirty micrograms of each sample was subjected to SDS-page electrophoresis gel, and then to Western blot analysis. Skeleton protein β-actin was selected as internal reference of cytoplasmic protein, histone H3 protein was selected as internal reference of nucleus protein. The results indicated that β-catenin protein levels in the cells stimulated by LiCl were significantly increased than those in un-stimulated cells, suggested that LiCl activated Wnt/β-catenin signaling pathway (**Figure 4**). In addition, β-catenin protein levels in the nucleus and cytoplasm of P1 infected cells were significantly decreased compared with those in mock infected cells stimulated by LiCl (**Figure 4**) demonstrated that P1 infection prevented β-catenin entering into the nucleus and abolished the activation of Wnt/β-catenin signaling stimulated by LiCl.

#### P1 Inactivates TCF/LEF Promoter Activity

**FIGURE 5:**
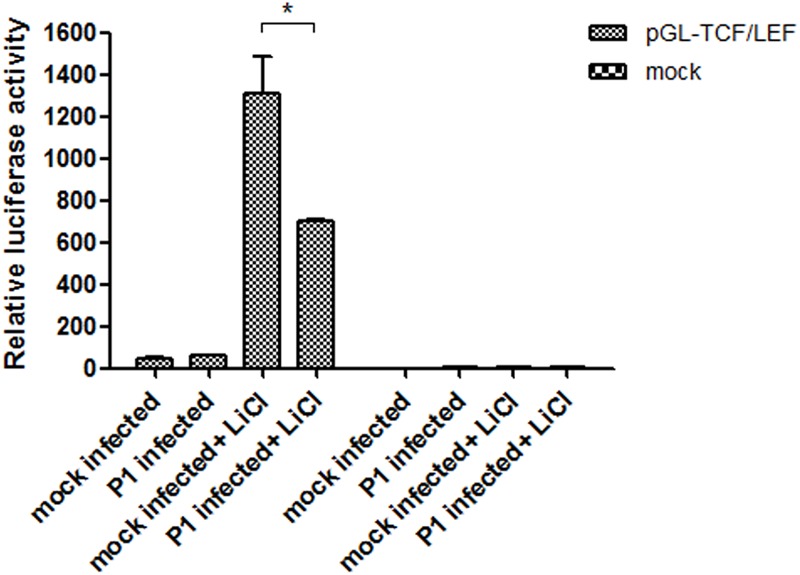
P1 inactivates TCF/LEF promoter activity. Cells in 24-well plates were transfected with either 1 μg pGL-TCF/LEF or pGL6 together with 0.1 μg pRL-TK. At 24 h post-transfection, the cells were infected with P1 infectious virus or ST cell lysate. At 24 hpi, cells were treated with 70 mM LiCl or not 24 h prior to harvest for luciferase assay analysis. The values were normalized with respect to Renilla luciferase activity. The results were indicated as fold-change of relative luciferase activities compared with the mock-treated group. All assays were repeated at three times, and the results are representative of one independent experiment. Statistical data were analyzed by one-way analysis of variance (^∗^*P* < 0.05). All data are expressed as the mean ± SD.

After transfection of ST cells with the pGL-TCF/LEF or the pGL6 control vector and pRL-TK, the cells were infected with P1 infectious virus or mock infected (ST cell lysate). At 24 hpi, 70 mM LiCl were added or not into culture. The groups were: pGL-TCF/LEF+P1+LiCl, pGL-TCF/LEF+P1, pGL-Basic6+P1+LiCl, pGL-Basic6+P1 and their mock infected groups. Luciferase assay results indicated that LiCl could increase the TCF/LEF promoter activities by near 1300-fold compared with its mock, and P1 infection significantly abolished the activation of TCF/LEF promoter by LiCl (*P* < 0.05) and the activity was reduced to half of pGL-TCF/LEF+LiCl group (**Figure 5**), further suggested that Wnt signaling pathway was inhibited by P1 infection *in vitro*.

### VP1 Inhibits Wnt Signaling Pathway *in Vitro*

#### VP1 Inhibits Downstream Components Expressions of Wnt Signaling Pathway

**FIGURE 6:**
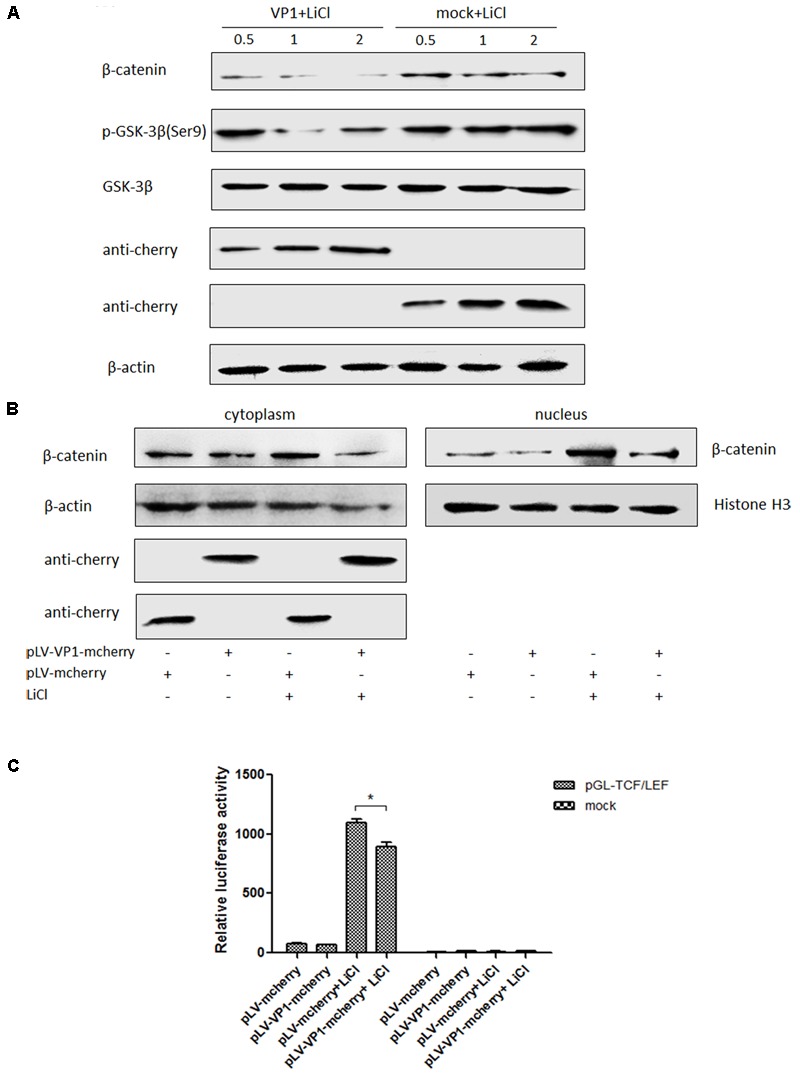
VP1 inhibits Wnt signaling pathway *in vitro*. **(A)** ST cells were transfected with pLV-VP1-mcherry or mock at indicated doses for 24 h, and treated with LiCl at a final concentration of 70 mM 24 h prior to harvest for Western blot analysis. Protein expressions of β-catenin and pGSK-3β/GSK-3β were analyzed. The protein expression levels of VP1-transfected cells are presented relative to that of mock-transfected cells. **(B)** ST cells were transfected with pLV-VP1-mcherry or mock for 24 h, LiCl at a final concentration of 70 mM were added into the culture or not 24 h prior to harvest for nucleus and cytoplasm separation and Western blot analysis. The bands were normalized with respect to β-actin (cytoplasm) and Histone H3 (nucleus). **(C)** pLV-VP1-mcherry or mock with pGL-TCF/LEF were co-transfected into ST cells 24 h prior to 70 mM LiCl stimulation or not; 24 h after the treatment by LiCl, luciferase assay was performed for TCF/LEF promoter activity analysis. The values were normalized with respect to Renilla luciferase activity. The results were indicated as fold-change of relative luciferase activities compared with the mock-treated group. All the assays were repeated at three times, and the results are representative of one independent experiment. Statistical data were analyzed by one-way analysis of variance (^∗^*P* < 0.05). All data are expressed as the mean ± SD.

VP1 is the structural protein of P1 virus, ST cells were transfected with pLV-VP1-mcherry and pLV-mcherry (mock) at 0.5, 1, 2 μg for 24 h and treated with LiCl at a final concentration of 70 mM 24 h prior to harvest for Western blot analysis. Endogenous β-catenin, GSK-3β and pGSK-3β protein expression levels were detected in VP1-infected ST cells at 24 h post-transfection and compared with those in uninfected cells. β-catenin expression level was significantly decreased in the VP1-transfected cells (*P* < 0.05) by band intensities scan software and the band intensities were normalized to β-actin (Supplementary Figure S2), while correspondent pGSK (Ser9) expression was decreased which weakened the inhibitory effect on its enzyme activity (**Figure 6A**), thus β-catenin accumulation was impaired and finally abolished the activation of the Wnt signaling pathway stimulated by LiCl. Consequently, it indicated that Wnt signaling pathway was inhibited by VP1 protein.

#### VP1 Prevents β-Catenin Entering Into the Nucleus

ST cells were transfected with pLV-VP1-mcherry or mock for 24 h, and then were treated with LiCl or not at a final concentration of 70 mM 24 h prior to harvest for nucleus and cytoplasm separation. Thirty micrograms of each sample was subjected to SDS-PAGE electrophoresis gel, and then to Western blot analysis. The results indicated that β-catenin protein levels in the cells stimulated by LiCl were significantly increased than those in un-stimulated cells, suggested that LiCl activated Wnt/β-catenin signaling pathway (**Figure 6B**). In addition, β-catenin protein levels in the nucleus and cytoplasm of VP1 transfected cells stimulated by LiCl were significantly decreased compared with those in mock transfected cells stimulated by LiCl (**Figure 6B**) demonstrated that VP1 protein prevented β-catenin entering into the nucleus and abolished the activation of Wnt/β-catenin signaling stimulated by LiCl.

#### VP1 Inactivates TCF/LEF Promoter Activity

ST cells were co-transfected with pLV-VP1-mcherry, pGL-TCF/LEF and pRL-TK or mock, respectively. At 24 hpi, cells were treated with 70 mM LiCl or not. Briefly, the groups were: pGL-TCF/LEF+VP1+LiCl, pGL-TCF/LEF+VP1, pGL-Basic6+VP1+LiCl, pGL-Basic6+VP1 or mock transfected groups. Luciferase assay results indicated that LiCl could activate TCF/LEF promoter, and VP1 protein significantly abolished the activation of TCF/LEF promoter stimulated by LiCl (*P* < 0.05) (**Figure 6C**), further demonstrated that Wnt signaling pathway was inhibited by VP1 of P1 *in vitro*.

## Discussion

*In vivo* studies have shown that porcine circovirus-like virus infected pigs displayed post weaning multi-systemic wasting syndrome (Wen et al., 2012d). In this study, we found that *in vivo* experiment, most downstream components of Wnt/β-catenin signaling pathway expression levels were significantly decreased in P1 infected piglets and mice which appeared weight loss, especially the mRNA expressions of c-myc, mmp2 and mmp9. The mmp2 and mmp9 expressions are collagen types. Ex-obese patients presented increased expression of mmp2; patients with extensive weight loss after bariatric surgery have decreased expression and activity of mmp9. These molecular changes may contribute for the formation of saggy skinfolds observed in these patients and impair wound healing (Prist et al., 2017). These results demonstrated that the weight loss induced by P1 infection was associated with the decreased expression of mmp2 and other downstream components of Wnt/β-catenin signaling. It is reported that c-myc is the target gene of Wnt/β-catenin signaling pathway, as well as the regulators of cells proliferation and a transcription factor that drives the synthesis of mRNAs and proteins (Daksis et al., 1994; Cole and Cowling, 2008; Ruggero, 2009; Wang et al., 2012; Lei et al., 2015). It drives us the suggestion that growth and development suppression caused by P1 may due to down-regulation of mmps and c-myc expressions.

LiCl guaranteed the Wnt signaling activated, thus clarified the P1’s effects on the Wnt signaling pathway. In this study, the viral loads were high in part of the mice and in which activation of Wnt signaling pathway by LiCl was abolished by P1 infection. However, the function of LiCl was limited in mice, Wnt signaling pathway components mmps increased significantly only in some tissues. Even though the function of LiCl was restrained, it still worked. The inhibitory effect of P1 was more obvious by LiCl stimulation compared with mock infected group with LiCl stimulation. In the pig experiment, the Wnt signaling activated piglets challenged by P1 virus displayed PMWS, other than the viral loads distribution in the different tissues, the lungs displayed thickening of the alveolar walls, decreased alveolar space, and increased amounts of inflammatory exudate. It was interesting that viral loads were quite low in lymph nodes and quite high in livers which were quite different from the PMWS caused by PCV2. However, weight loss is the typical clinical symptom of PMWS caused by PCV2 or P1. Wnt/β-catenin signaling pathway is associated with the young animal growth. In our study, we proved that Wnt/β-catenin signaling pathway was inhibited *in vitro* and *in vivo* infected by P1, while none infected animal didn’t show weight loss or Wnt/β-catenin signaling pathway inhibition. We would like to see the reverse weight gain by using LiCl in the mice challenged by P1. However, the result didn’t achieve our expectation. Of course, growth development is a complicated process contains varieties of signalings, in now stage, we only confirmed that P1 infection was associated with Wnt/β-catenin signaling pathway suppression, the mechanism that weight loss caused by P1 infection is related to Wnt/β-catenin signaling suppression would be studied in the near future.

Subsequently *in vitro* study, similar with our previous study *in vivo*, we found that P1 infection downregulated the mRNA expression of mmp2 and protein expression of β-catenin *in vitro*. However, not all the mRNA expressions of downstream components of β-catenin/Wnt signaling pathway were down-regulated, indicating P1 also activated or inactivated other pathways that might influence or cross react with the Wnt signaling pathway, more studies would be performed to prove that the other possible signaling pathways might affect β-catenin/Wnt signaling pathway. In addition, P1 disrupted the entry of β-catenin from cytoplasm into nucleus, inactivated TCF/LEF promoter activity, which downregulated the expressions of targeted genes (mmps and c-myc) which were consistent with the results *in vivo* experiment.

VP1 is the vital structural protein and capsid protein of P1 virus. Little was known about this capsid protein. Phosphorylation of Ser9 of GSK3β inhibits its catalytic activity (Li and Paudel, 2007; Zheng et al., 2007; Hu et al., 2008; Inkster et al., 2010; Niehrs and Acebron, 2012; McCubrey et al., 2014; Reischmann et al., 2015). VP1 protein inhibited Wnt/β-catenin signaling pathway by downregulating p-GSK3β (Ser9)/GSK3β and β-catenin protein levels in ST cells, inactivated the TCF/LEF promoter, and prevented β-catenin from entering into nucleus. However, the possibility that other ORF encoded proteins of P1 suppressing Wnt/β-catenin signaling pathway cannot be excluded since the inhibitory effect of VP1 on the Wnt signaling pathway was not as significant as that of P1 infection.

Taken together, all the above results suggested that the P1 infection might be associated with the down regulation of Wnt/β-catenin signaling pathway *in vivo* and *in vitro*.

## Author Contributions

KH and XZ designed the experiments. XZ performed most of the experiments in this study and analyzed the data. LW helped and analyzed the data and revised the manuscript. MQ, SS, and WW provided the cells and helped to perform the animal experiments. QX, YH, and CL helped to prepare the experiments.

## Conflict of Interest Statement

The authors declare that the research was conducted in the absence of any commercial or financial relationships that could be construed as a potential conflict of interest.
