# Supplementary material for: Porcine Circovirus-Like Virus P1 Inhibits Wnt Signaling Pathway in Vivo and in Vitro
Source: Front Microbiol. 2018 Mar 12;9:390. doi: 10.3389/fmicb.2018.00390 (PMC5857601; doi:10.3389/fmicb.2018.00390)
Supplement: Supplementary file 1 [file Data_Sheet_1.DOCX]

**Porcine circovirus-like virus P1 inhibits Wnt signaling pathway in vivo and in vitro**

**
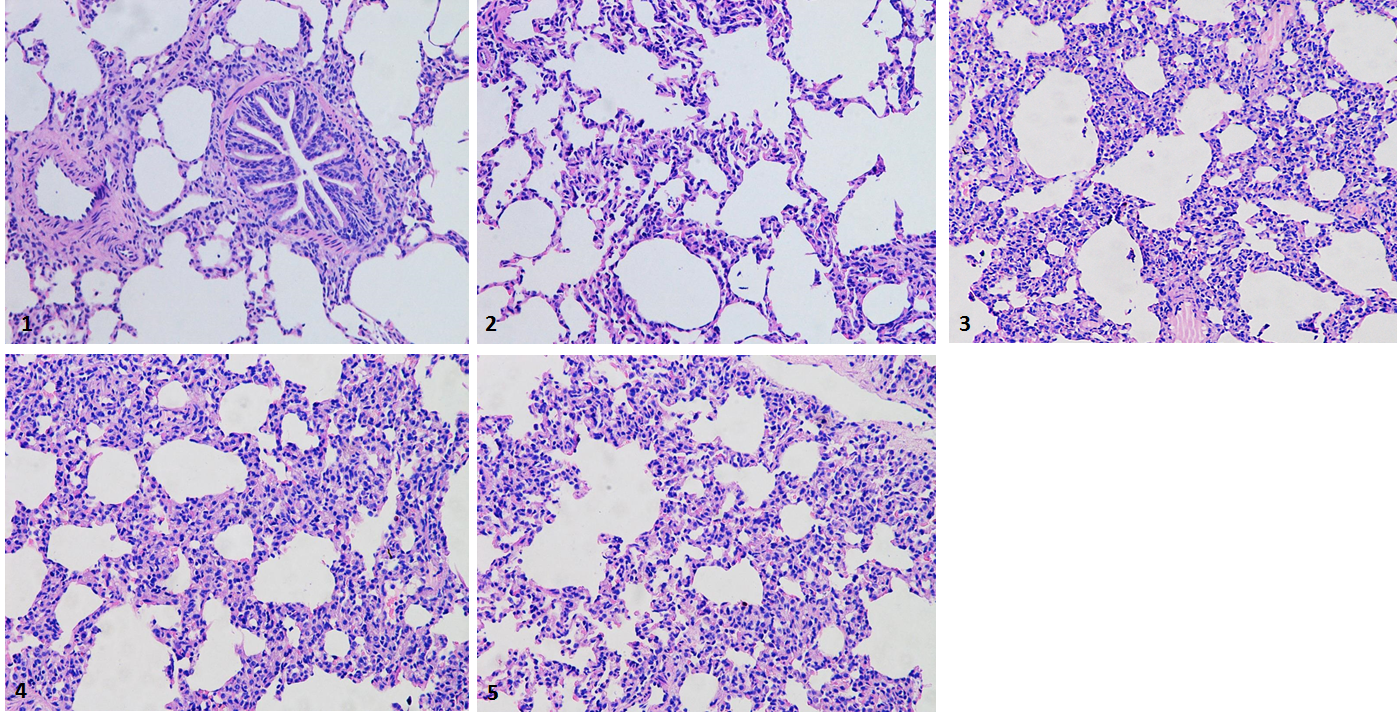
**

**Fig.S1** **Lung lesion sections of PMWS piglets**

The lung lesion of piglets displayed PMWS showed thickening of the alveolar walls, decreased alveolar space, and increased amounts of inflammatory exudate. Note interstitial pneumonia characterized by greatly thickened interlobular septum and alveolar septal thickening, a small number of alveolar septum fractures, some expansion of the alveoli filled with red blood cells. HE staining was used for all panels. Magnification. × 40.


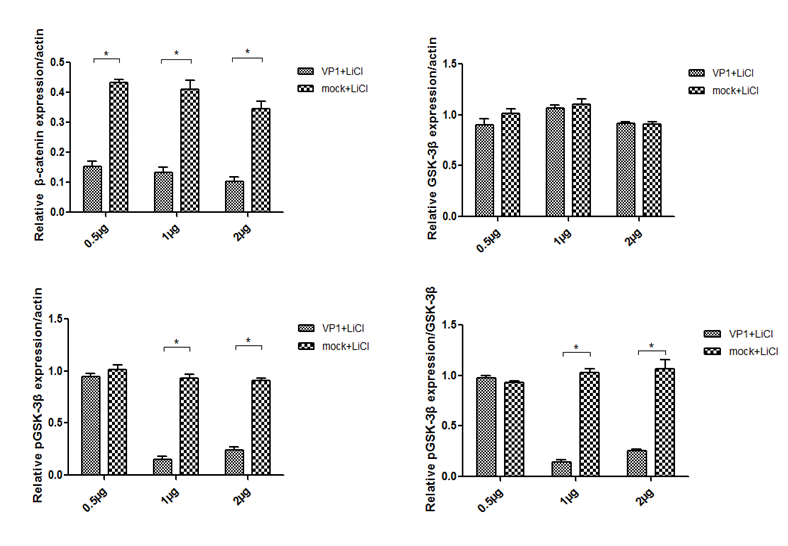


**Fig.S2 Protein band intensities comparison**

| **ADWG (g)** | | | | | |
| --- | --- | --- | --- | --- | --- |
| **2wpi compared with 0wpi** | | **3wpi compared with 0wpi** | | **4wpi compared with 0wpi** | |
| **control** | **challenge** | **control** | **challenge** | **control** | **challenge** |
| **0.00651±0.00189** | **0.00629±0.00233** | **0.00539±0.00145** | **0.00470±0.00152** | **0.00535±0.00171** | **0.00474±0.00138** |

The statistical difference was analyzed by band scan and the band intensities were normalized to that of β-actin. Statistical data were analyzed by one-way analysis of variance. (*, P<0.05). All data are expressed as the mean ± SD.

**Table.S1 Average day gain at each week in mice experiment**

ADWG indicates relative average daily weight gains in mice experiment from 0dpi to 28dpi. Data are presented as the mean ± SD.
